# Trends in Clinical Pharmacist Integration in Family Medicine Residency Programs in North America

**DOI:** 10.3390/pharmacy8030126

**Published:** 2020-07-24

**Authors:** Jennie B. Jarrett, Jody L. Lounsbery

**Affiliations:** 1 Department of Pharmacy Practice, Chicago College of Pharmacy, University of Illinois, Chicago, IL 60612, USA; 2 Department of Pharmaceutical Care & Health Systems, College of Pharmacy, University of Minnesota, Minneapolis, MN 55455, USA; loun0015@umn.edu

**Keywords:** pharmacist, medical education, family medicine, graduate medical education, interprofessional

## Abstract

(1) **Objective**: To determine the change in prevalence of clinical pharmacists as clinician educators within family medicine residency programs (FMRPs) in North America and to describe their clinical, educational and administrative scope over time. (2) **Methods**: A systematic review of the literature was performed starting with an electronic search of PubMed and Embase for articles published between January 1980 and December 2019. Studies were included if they surveyed clinical pharmacists regarding their clinical, educational, or other roles in FMRPs in the United States or Canada. The primary outcome was the change in prevalence of clinical pharmacists in North America. Secondary outcomes included: demographic information of clinical pharmacists, change in the prevalence in Canada and United States, and descriptions of clinical services, educational roles, and other activities of clinical pharmacists within FMRPs. (3) **Results**: Of the 65 articles identified, six articles met the inclusion criteria. The prevalence of clinical pharmacists as clinician educators in FMRPs in North America has grown from 24% to 53% in the United States (U.S.) and from 14% to 47% in Canada over the study period. The clinical and educational roles are similar including: the direct patient care, clinical education, and interprofessional education and practice. (4) **Conclusion**: The prevalence of clinical pharmacists in FMRPs is growing across North America. Clinical pharmacists are highly educated and trained to support these clinician educator positions. While educational roles are consistent, clinical pharmacists’ patient care roles are unique to their clinical site and growing.

## 1. Introduction

The American College of Clinical Pharmacy outlined eight standards of practice for clinical pharmacists, including qualifications, process of care, documentation, collaborative team-based practice and privileging, professional development and the maintenance of competence, professionalism and ethics, research and scholarship, and other responsibilities. These other responsibilities may include the roles of educators, clinical preceptors, mentors, administrators, and policy developers. Based on these standards, clinical pharmacists are educated and trained professionals who work in direct patient care environments. Clinical pharmacists use a patient care framework, the Pharmacist Patient Care Process, to identify, assess, evaluate, and monitor patients’ medication-related needs. Clinical pharmacists collaborate directly with other healthcare professionals to provide care for patients [1,2]. Within training programs like family medicine residency programs (FMRPs), clinical pharmacists have the opportunity to display their role as clinician educators. Clinical educators are practitioners who are also dedicated to teaching and developing themselves as educators [3].

Clinical pharmacists have been clinician educators in family medicine residency programs (FMRPs) for several decades, with the first account of their roles documented in 1977 [4]. Clinical pharmacists’ roles within FMRPs have been further described, and family medicine physician perceptions of their integration in both the United States and Canada have been positive. Physicians reported having clinical pharmacists integrated into their practices which resulted in positive effects on patient care, meaningful contributions to knowledge, and an increased understanding of interprofessional team practices [5,6,7,8,9].

Pharmacy education and training in North America has evolved over the past several decades to support direct patient care, interprofessional education, and collaborative practices. The latest standards in the United States from the Accreditation Council for Pharmacy Education incorporates the Institute of Medicine recommendations for the education of all healthcare professionals [10]. Attributes these pharmacists should possess upon graduation include competencies to meet the needs of contemporary practice such as: provide patient-centered care, work in interprofessional teams, employ evidence-based practices, apply quality improvement methods, and use informatics [11]. The most recent standards from the Canadian Council’s Accreditation of Pharmacy Programs also mirror these competencies [12]. Post-graduate pharmacy residency training in both the United States and Canada embrace these competencies as well to train pharmacy graduates in the additional skills necessary for these unique patient care and educational positions [13,14,15]. This shift in focus to address the needs of contemporary practices, including interprofessional education and collaborative practices, has likely influenced the role of the clinical pharmacist within FMRPs.

The primary objective of this review is to determine the change in prevalence of clinical pharmacists as clinician educators in FMRPs in North America over time. The secondary objective was to describe the clinical, educational, and administrative scope of these clinical pharmacists in FMRPs.

## 2. Materials and Methods 

This systematic review was performed in accordance with the preferred reporting items for systematic reviews and meta-analyses (PRISMA) guidance [16].

A researcher (J.L.L.) conducted an electronic search of PubMed and Embase for articles published between January 1980 and December 2019. The search was completed on 8 May 2020. Searches included keywords of the following terms: *family medicine residency, family practice residency, pharmacist*, and *pharmacy*. Broad terms, such as family medicine residency, were combined in strings with specific terms, such as pharmacist, for focused results. Search results were limited to the English language. Bibliographies of the included articles were reviewed for potential additional articles for meeting the criteria for inclusion. 

Studies were included in this review if they surveyed clinical pharmacists regarding their clinical, educational, or other roles in FMRPs in the United States or Canada. Studies were excluded if: (1) only abstracts could be obtained via library access at either the University of Minnesota or University of Illinois at Chicago; (2) the survey related to an intervention-based project or service; (3) the survey was not conducted nationally across either the U.S. or Canada; (4) it was not within an FMRP. Article citations and abstracts were downloaded into a text document for review. Authors (J.B.J. and J.L.L.) performed title and abstract screening independently. The title and abstract screening results were discussed between the authors (J.B.J. and J.L.L.) and inclusion/exclusion discrepancies were determined through consensus. 

The primary outcome was the change in prevalence of clinical pharmacists in North America. Secondary outcomes included: the demographic information of clinical pharmacists, change in prevalence in Canada and the United States, and descriptions of clinical service, educational roles, and other activities of clinical pharmacists within FMRPs. 

Descriptive statistics were used to calculate quantitative data not described explicitly by the study authors. A chi-squared statistical test was performed to determine the changes in prevalence of clinical pharmacists in FMRPs in the U.S. and Canada. A thematic analysis of qualitative information was completed systematically by coding data for the themes in practice and educational roles to summarize and describe clinical and educational activities [17]. 

## 3. Results

**Figure 1:**
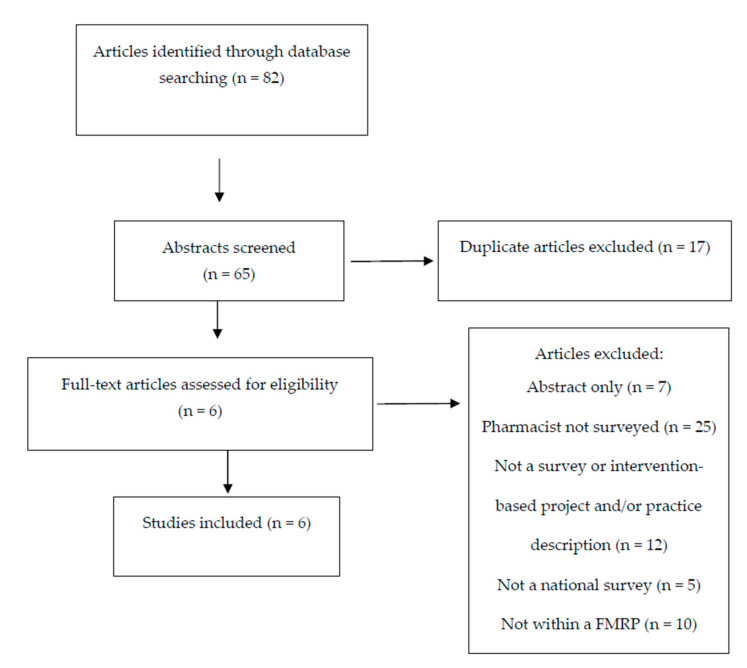
Flow diagram of the study.

Of the 65 unique articles identified in PubMed and Embase, six studies met the inclusion criteria for analysis in this review [18,19,20,21,22,23]. The flowchart for inclusion and exclusion is provided in Figure 1. Of the six studies included, four studies occurred in the United States [18,19,21,23] and two studies occurred in Canada [20,22]. All of the included studies used similar survey methodology, including contacting FMRP program directors or their program administrators to identify the clinical pharmacists practicing within each FMRP. Each of the included studies used unique, researcher derived surveys with varying areas of focus for collecting data on pharmacists within FMRPs as noted in the data below. Surveys were not available for analysis. 

### 3.1. Prevalence

The prevalence of clinical pharmacists in FMRPs has grown from 24% in 1990 to 53% in 2015 (95% CI 21.2–34.7; *p* < 0.001) in the United States [19,21,23] and from 14% in 1994 to 47% in 2009 (95% CI 17.6–45.1; *p* < 0.001) in Canada [20,22]. Table 1 describes the number of programs surveyed, program response rates, the number of programs with clinical pharmacists, and pharmacist survey response rates. The demographics of clinical pharmacists in FMRPs from 1983 to 2015 are described in Table 2. Overall, clinical pharmacists were young (<40 years old) with a PharmD degree, residency training, and had an appointment in a college/school of pharmacy or medicine.

### 3.2. Clinical and Educational Scope

Within the Unites States, clinical pharmacists’ time spent in direct patient care roles rose from 36% in 1990 to 53% in 2015, while time spent in teaching roles decreased from 43% in 2000 to 32% in 2015 [19,21,23]. The time clinical pharmacists in the United States reported spending on various areas within the FMRPs is shown in Table 3. The time Canadian clinical pharmacists spent was not described in the literature. 

Regarding patient care activities, clinical pharmacists consistently provided patient education [18,19,20,22] and drug information [18,19,20,22,23]. Patient care services were reported in both inpatient [18,20,21,23] and outpatient settings [19,20,21,22,23]. The types of patient care activities reported include: inpatient rounding, direct patient care in outpatient practice, chart reviews, patient assistance programs, pharmacokinetic drug monitoring, nursing home visits and discharge counseling. 

Clinical pharmacists within FMRPs have consistently provided clinical education through drug information [18,19,20,22,23] and indirect care activities, such as precepting, consults, and/or chart reviews [18,19,20,21,22,23]. Educational activities also often included formal teaching, such as didactic presentations and conferences [18,19,21,22,23] and also the noted facilitation of specific residency rotation for family medicine residents [19,21,23]. The learners consisted of family medicine residents [18,19,20,21,22,23] and pharmacy students [18,19,20,21,23]. Other interprofessional learners included medical students [19,20,21,23], nurses and nurse practitioners [18,19,20,21,23]. Pharmacy residents were first reported as learners in 2000 [21,23]. 

## 4. Discussion

Interprofessional education and training via clinical pharmacists as clinician educators within FMRPs is well established. This research sought to define the trends in the prevalence and clinical and educational scope over time within FMRPs in North America. Clinical pharmacists within FMRPs have grown significantly over the last 40 years in both the United States and Canada. The growth in integration of clinical pharmacists appears to have been through an expansion of their clinical and interprofessional teaching roles, with reductions in administrative and research time.

A swift growth in pharmacy residency training positions has supported this growth of clinical pharmacists within FMRPs. Post-graduate training for pharmacists has occurred since the 1930s with the official classification of residency training in the 1960s [24]. Pharmacy residency programs are delineated as post-graduate year 1 (PGY1) programs, which are general hospital practice-focused, or post-graduate year 2 (PGY2) programs, which are specialized in one area such as ambulatory care or critical care. Accredited PGY1 pharmacy residency programs have grown exponentially from roughly 1600 positions in 2007 to 3924 positions in 2020 [24,25]. Similarly, accredited ambulatory care PGY2 pharmacy residencies, the pharmacy specialty most congruent to family medicine, has grown from 62 positions in 2013 to 187 positions in 2020 [25]. In addition to clinical practice training and experience within residency training, many pharmacy residencies provide teaching experiences and faculty development [26,27]. The rapid growth of pharmacy residency programs for the training of clinical skills with an incorporation of teaching and faculty development has encouraged and supported the growth of competent clinician educators as pharmacists within FMRPs. Recently, the Accreditation Council for Graduate Medical Education (ACGME) expanded their medical residency faculty definition to include non-physician members, such as clinical pharmacists [28]. The scholarship, teaching, and education of clinical pharmacists within FMRPs now supports the overall FMRP faculty program requirements for accreditation. 

Physician–pharmacist collaborative practices improve patient care outcomes and are cost effective [29]. Yet, barriers exist to the full integration of pharmacists into the care team, including perceptions of knowledge deficits, limited experience working with pharmacists, and communication challenges [30]. The integration of clinical pharmacists within FMRPs can invalidate many of these perceived barriers early in a physician’s career, building the foundation for long-term, progressive incorporation of team-based care to improve patient outcomes, patient satisfaction, and provider satisfaction. Specifically, integrating clinical pharmacists may also help family medicine physicians and other members of the health care team meet the quadruple aims of improving population health, improving the patient’s experience of care, reducing the per capita cost of health care, and improving the provider experience [31,32]. Pharmacists within FMRPs should share explicit information regarding their education, training, and benefits to their roles with physician residents and faculty to remove the perceived bias and implicit attitudes. 

There are limitations to this systematic review of the literature. Each of the studies included in this review used different surveys for the collection of its data, making accurate comparisons challenging. Additionally, there are significant differences in healthcare and health-systems in the United States compared to Canada. While growth in team-based care is universal between the two countries, there are financial confounders in the U.S. related to the privatization of healthcare that limits the feasibility of the incorporation of clinical pharmacists within FMRPs. 

The prevalence of clinical pharmacists within FMRPs in North America is growing. The education and training changes support clinical pharmacists as valuable clinicians for direct patient care and faculty members within FMRPs. The standardization of the integration of pharmacists within FMRPs supports training resident physicians to collaborate with pharmacists throughout their careers to improve patient outcomes in their practice. 

## 5. Conclusions

The prevalence of clinical pharmacists in FMRPs is growing across North America. Clinical pharmacists are highly educated and trained to support these clinician educator positions. 

## Figures and Tables

**Table 1:** Clinical pharmacists in family medicine residency programs in North America from 1983 to 2015.

Survey Year (Country)	Total Number of Programs, n	Programs with Responses Obtained, n	Programs with Clinical Pharmacists, n	Programs with Pharmacists out of Programs with Responses, %	Pharmacist Survey Response Rate, %
2015 [23] (U.S.)	480	396	208	52.5 (208/396)	56.1 (142/253)
2009 [22] (Canada)	158	86	40	46.5 (40/86)	80.0 (32/40)
2000 [21] (U.S.)	579	555*	155	27.9 (155/555)	74.7 (130/174)
1994 [20] (Canada)	82	58	8	13.8 (8/58)	100.0 (9/9)
1990 [19] (U.S.)	381	325	79	24.3 (79/325)	NR
1983 [18] (U.S.)	386	NR	68	NR	72.1 (49/68)

Note: For the pharmacist survey response rate, the denominator represents the number of pharmacists identified from the programs with pharmacists. The numerator indicates the number of pharmacists responding to the survey. *Number was extrapolated from published data in each article, and was based on the calculation from response rate. U.S. = United States; NR = not reported.

**Table 2:** Demographics of clinical pharmacists in family medicine residency programs in North America from 1983 to 2015*.

Characteristic	1983 [18] (US), n = 49	1990 [19] (US), n = 80	1994 [20] (Canada), n = 9	2000 [21] (US), n = 130	2009 [22] (Canada), n = 32	2015 [23] (US), n = 142
Age, years	13 (27%) <30	34.6 (range 24–51)	“Most” were 30–40	36.5 ± 8.2 (range 25–59)	78% <45	38.5 ± 9.6 (range 26–67)
	29 (59%) 30–40					
	4 (8%) 41–50					
	3 (6%) 51–60					
	0 (0%) >60					
Gender						
Male	37 (76%)	68%	1 (11%)	46%	36%	43 (30%)
Female	12 (24%)	32%	8 (89%)	54%	65%	99 (70%)
Degree						
PharmD	67%	85%	1 (11%)	89%	76%	138 (97%)
Residency	53%	68%	5 (56%)	69%	NR	104 (86%)
Academic appointment						
C/SOP	28 (57%)	61 (76%)	NR	80%	NR	105 (74%)
C/SOM	19 (39%)	29 (36%)	NR	52%	NR	69 (49%)

*Reporting in the table varies based on how the data were reported in the studies. Underlined numbers were calculated based on the published data in each article. PharmD = doctor of pharmacy; C/SOP = college/school of pharmacy; C/SOM = college/school of medicine.

**Table 3:** Clinical pharmacist percentage of time spent within the United States family medicine residency programs.

	1990 [19]	2000 [21]	2015 [23]
Patient Care, %	36	37	53
Teaching, %	35	43	32
Research/Scholarship, %	12	12	8
Administration, %	NR	12	6

NR = not reported.
